# EarGait: Estimation of Temporal Gait Parameters from Hearing Aid Integrated Inertial Sensors

**DOI:** 10.3390/s23146565

**Published:** 2023-07-20

**Authors:** Ann-Kristin Seifer, Eva Dorschky, Arne Küderle, Hamid Moradi, Ronny Hannemann, Björn M. Eskofier

**Affiliations:** 1 Machine Learning and Data Analytics Lab (MaD Lab), Department Artificial Intelligence in Biomedical Engineering (AIBE), Friedrich-Alexander-Universität Erlangen-Nürnberg (FAU), 91052 Erlangen, Germany; eva.dorschky@fau.de (E.D.); arne.kuederle@fau.de (A.K.); hamid.moradi@fau.de (H.M.); bjoern.eskofier@fau.de (B.M.E.); 2 WS Audiology, 91058 Erlangen, Germany; ronny.hanneman@wsa.com

**Keywords:** earables, gait analysis, gait event detection, inertial sensor, SSA, wearables

## Abstract

Wearable sensors are able to monitor physical health in a home environment and detect changes in gait patterns over time. To ensure long-term user engagement, wearable sensors need to be seamlessly integrated into the user’s daily life, such as hearing aids or earbuds. Therefore, we present EarGait, an open-source Python toolbox for gait analysis using inertial sensors integrated into hearing aids. This work contributes a validation for gait event detection algorithms and the estimation of temporal parameters using ear-worn sensors. We perform a comparative analysis of two algorithms based on acceleration data and propose a modified version of one of the algorithms. We conducted a study with healthy young and elderly participants to record walking data using the hearing aid’s integrated sensors and an optical motion capture system as a reference. All algorithms were able to detect gait events (initial and terminal contacts), and the improved algorithm performed best, detecting 99.8% of initial contacts and obtaining a mean stride time error of 12 ± 32 ms. The existing algorithms faced challenges in determining the laterality of gait events. To address this limitation, we propose modifications that enhance the determination of the step laterality (ipsi- or contralateral), resulting in a 50% reduction in stride time error. Moreover, the improved version is shown to be robust to different study populations and sampling frequencies but is sensitive to walking speed. This work establishes a solid foundation for a comprehensive gait analysis system integrated into hearing aids that will facilitate continuous and long-term home monitoring.

## 1. Introduction

Physical mobility can be impaired by various chronic health conditions such as cardiovascular diseases, neurodegenerative disorders, physical frailty, or cognitive functioning [1]. Therefore, monitoring mobility, and specifically gait, gives an insight into the current health condition of a human. Home-monitoring systems are gaining popularity as studies have shown that in-lab assessments capture only a small portion of the variance observed in home environments [2].

Wearable sensors, such as Inertial Measurement Units (IMUs), enable continuous long-term measurements and are often used for home monitoring; however, serious concerns about their ease of use and suitability for daily use remain [3]. One major problem of existing approaches is that they introduce an additional device that needs to be worn, operated, and charged by the user. To sustain long-term user engagement, wearable devices should ideally be seamlessly integrated into a device or garment that is already part of the user’s daily life [4,5]. For a large portion of the elderly population, hearing aids (HA) are a promising device for integration. Interest in earables for ubiquitous sensing is growing as their small and lightweight form allows them to be worn for prolonged periods throughout the day [6]. Hearing aids were originally developed for the isolated purpose of sound amplification but are now equipped with motion sensors that could be used for physical health tracking, transforming an HA into a multi-functional device [7]. Moreover, a hearing-aid-embedded health tracker would be particularly useful, as hearing loss is often associated with a decline in physical health [8].

Rahme et al. [9] proposed the first idea of how motion sensors could be used for physical health assessment by presenting an HA integrated fall detector and step counter. Not only the number of steps but gait parameters in general are of great interest for mobility assessment. Gait is considered the most important type of mobility, and changes in gait patterns can be linked to various deficits such as dysfunctional mobility, fall risk, cognitive decline, or mental impairments [10,11,12]. Gait impairments can be characterized by changes in various gait measures, such as cadence, gait speed, and stride length [13].

For home-monitoring gait analysis systems, single sensor systems are preferred [14], having the advantage of capturing gait-related patterns from both limbs simultaneously. While Lindemann et al. [15] suggested that head-worn sensors could provide better insights into physical impairments than sensors located at the hip or wrist, head-worn sensors remain poorly investigated [16,17].

A few papers have specialized in gait classification using head-worn IMUs. Burgos et al. [18] introduced a machine-learning-based classifier to distinguish walking and running, and Atallah et al. [19] used an ear-worn sensor to detect gait impairment. A posture identification system for detecting different walking styles of patients with knee osteoarthritis was presented in [20]. However, classical gait analysis pipelines give a comprehensive insight into gait patterns by extracting spatiotemporal parameters, for instance, step length or gait speed. Most existing ear- or head-worn gait analysis systems focus only on a single gait parameter, such as gait speed [21] or gait cycle duration [22]. A comprehensive pipeline for gait analysis using ear-worn sensors that includes multiple gait parameters is lacking to date.

From an algorithmic perspective, a crucial aspect of such a gait analysis pipeline is the segmentation of recorded data into meaningful portions, such as individual strides. A stride is defined by the initial contact (IC) of one foot until the next IC of the same foot. Consequently, many gait analysis pipelines contain a gait event detection algorithm to detect initial and terminal contact (TC) events. In particular, most step and stride length algorithms directly rely on the detection of gait events [23,24,25], underscoring the significance of a reliable and accurate gait event detection algorithm as a fundamental component in comprehensive gait analysis pipelines. To our best knowledge, two algorithms have been described in the literature for gait event detection using ear-worn sensors [26,27]. Jarchi et al. [26] proposed an algorithm based on Singular Spectrum Analysis (SSA). They validated their method at different speeds with an instrumented treadmill. Diao et al. [27] proposed a similar algorithm and validated their method using multiple lower-body-worn IMUs. The algorithm was assessed for healthy young participants and postoperative patients. Both approaches are capable of estimating temporal gait parameters with high accuracy. However, the algorithms have not been validated against the gold standard for gait analysis, an optical motion capture (OMC) system. Moreover, the sensitivity and the distinction between ipsi- and contralateral steps have not been evaluated yet. The correct distinction between gait events from the left and the right foot is crucial for the computation of different stride-by-stride measures, including gait asymmetry [28], which is a promising marker for certain pathological gait conditions [29].

**Figure 1:**
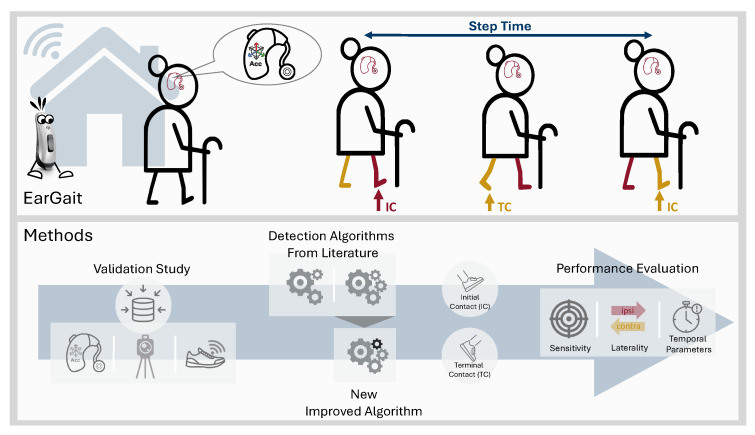
Overview figure showing the contributions of the paper and the EarGait package [30]. The gait event detection algorithms detect initial contact (IC) and terminal contacts (TC). Red and yellow were used to differentiate between the leg on the same side as the hearing aid (ipsilateral) and the opposite side (contralateral).

To address these limitations, we present the following contributions, which are also illustrated in Figure 1:

1. As the first step in a comprehensive gait analysis pipeline for ear-worn sensors, we present a comparative validation for different gait event detection methods [26,27] using an optical motion capture system as a reference. Algorithms were evaluated in terms of detection rate, laterality determination, and temporal parameters.
2. To enhance the accuracy of the laterality determination of gait events, we propose an improved version of the algorithm by Diao et al. [27].
3. The calculated temporal gait parameters are compared with respect to different walking speeds, study cohorts, and sampling frequencies.
4. We published an open-source Python package called *EarGait* providing functions for gait analysis using ear-worn sensors, including data loading functionalities, event detection algorithms, and temporal parameter estimation [30].

## 2. Materials and Methods

### 2.1. Data Collection

We recorded a data set containing 27 young and 21 elderly participants (Table 1). All participants were healthy with no limiting physical health conditions. The participants gave written informed consent prior to the recording, and the study was approved by the local ethics committee (Friedrich-Alexander-Universität Erlangen-Nürnberg, Erlangen, Germany) Re-No. 106_13B. It took place at the Motion Capture Lab of the Institute of Applied Dynamics (LTD - Lehrstuhl für Technische Dynamik, Friedrich-Alexander Universität Erlangen-Nürnberg).

**Figure 2:**
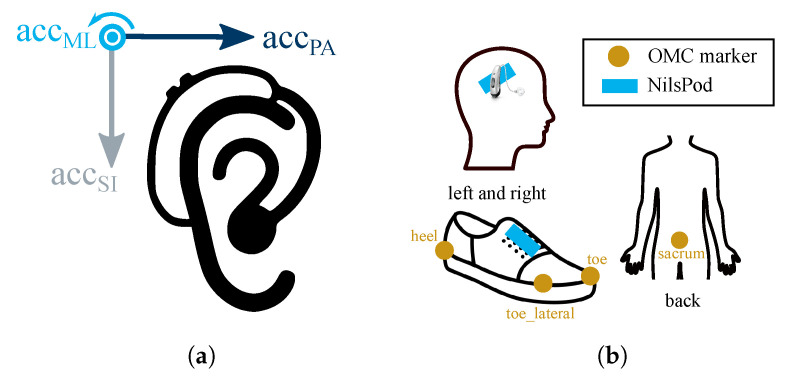
The sensor setup included (**a**) two hearing aids with integrated IMU and (**b**) optical motion capture (OMC) markers and NilsPod IMU sensors. The axes of the coordinate system are defined by the main anatomical axes, namely medial to lateral (acc_ML_), posterior to anterior (acc_PA_), and superior to inferior (acc_SI_).

Participants were equipped with a pair of HAs (left and right side) with an integrated IMU (3D accelerometer, ±16 g; 3D gyroscope, ±2000°/s; $f_{s}=200\;\mathrm{Hz}$). HAs are shown in Figure 2a and were provided by WS Audiology (Erlangen, Germany and Lynge, Denmark), the amplification was turned off. We used an OMC system (Qualisys) with ten infrared cameras (Oqus 700+, $f_{s}=200\;\mathrm{Hz}$, Wand and L-frame calibration: residual error and standard deviation <1 mm) as a reference system. Participants were equipped with seven retro-reflective markers, as shown in Figure 2b. Additionally, NilsPod IMU sensors (Portabiles GmbH, Erlangen, Germany; 3D accelerometer, ±16 g; 3D gyroscope, ±2000°/s; $f_{s}=204.8\;\mathrm{Hz}$) were attached to the shoes and above the ears. All NilsPod sensors were synchronized using a wireless synchronization protocol [31]. Furthermore, we synchronized the OMC to the NilsPod sensors via an analog trigger. We calibrated each IMU sensor (hearing aid integrated IMUs and NilsPod IMUs) prior to the recording using the calibration method introduced by Ferraris et al. [32]. Participants walked a straight path of 8 m within the calibrated capture volume, turned on the spot and walked back. For each speed, six of these trials were recorded with a short break between each trial. Each participant walked at three different self-selected speeds (normal, fast, and slow), resulting in a total of 18 trials per participant.

### 2.2. Reference Gait Parameters

**Figure 3:**
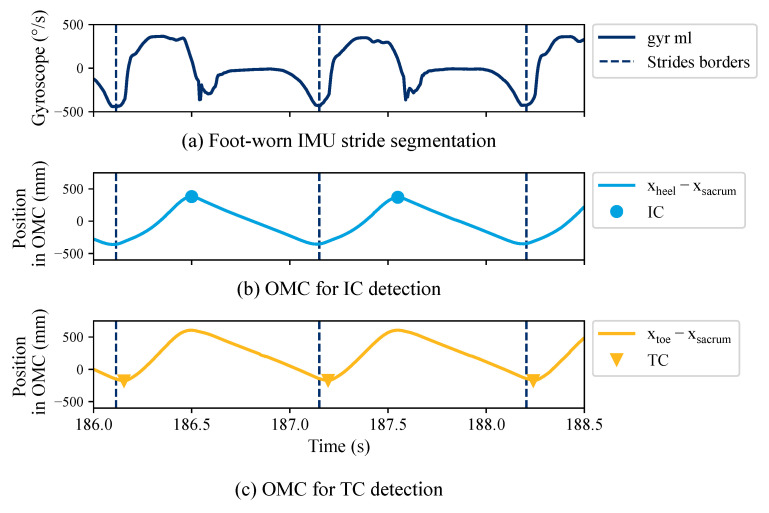
Reference gait events were estimated in two steps. (**a**) First, foot-worn IMU data were used to segment the region of interest, i.e., individual strides. Ref. [33] was applied to determine stride borders, followed by manual quality control. (**b**,**c**) Within each region of interest, gait events (initial contact–IC, terminal contact–TC) were estimated using the optical motion capture (OMC) system and the coordinate-based algorithm by Zeni et al. [36]. The example shows signals and gait events for the left side. The right side is not shown for visual clarity.

We applied a two-step approach to estimate gait events for the OMC systems, which is considered as the gold standard for gait analysis (Figure 3). First, we used the foot-worn IMU data to segment individual strides considered as regions of interest. Strides were segmented by a dynamic time-warping algorithm [33] followed by a manual quality control using the MaD GUI [34]. Turning strides were excluded from the data set. Second, within each region of interest, we derived the gait events using the OMC. Based on the recommendation by Bruening et al. [35], the coordinate-based gait event detection algorithm by Zeni et al. [36] was implemented. $IC_{OMC}$ and $TC_{OMC}$ are estimated by finding local minima or maxima within each region of interest:

(1a) $$\begin{array}{cc} IC_{OMC} & =max(x_{heel}-x_{sacrum}) \end{array}$$

(1b) $$\begin{array}{cc} TC_{OMC} & =min(x_{toe}-x_{sacvrum}), \end{array}$$

where $x_{heel}$, $x_{toe}$, and $x_{sacrum}$ refer to the *x* coordinate (pointing in the direction of walking) of the heel, toe, and sacrum marker, respectively (Figure 2b). The original approach by Zeni et al. [36] applies the peak/valley detection to the entire gait signal. We applied the algorithm within the hand-labeled regions of interest to reduce the number of falsely detected peaks and ensure exactly one IC and one TC event for each stride.

### 2.3. Gait Event Detection (GED)

HA sensor data were transformed into a coordination system defined by the main anatomical axes, namely ML (medial to lateral), SI (superior to inferior), and PA (posterior to anterior) (Figure 2a) as further described in [37]. In this coordinate system, the data of left and right HA are mirrored images of each other. As a result, the same signals can be expected for the same anatomical movement independent of the sensor’s placement (left or right ear). This is advantageous for any kind of event detection on signals because the same algorithm can be applied to both sides without further coordinate transformations. Additionally, the data were aligned with the gravity vector. In contrast to the NilsPod sensors, the HA recordings were not synchronized with the reference OMC system. HA and ear-worn NilsPod IMU data were synchronized using cross-correlation to obtain walking intervals.

Two essential gait events need to be detected in order to segment individual strides and estimate temporal gait parameters: initial contact (IC) and terminal contact (TC). Ipsilateral (IL) and contralateral (CL) are used to distinguish between gait events of the same and opposite foot in relation to the sensor’s position. Both algorithms, *Jarchi* and *Diao Original*, apply an SSA to extract dominant oscillations associated with the repetitive gait pattern. An SSA is a model-free technique to decompose a signal into multiple orthogonal components, including slow varying trends, oscillations, and unstructured noise [38]. For ear-worn sensors, the slow trend is associated with head movements [26].

The main difference between the two approaches is the selected axes used for detecting gait events. Furthermore, the *Jarchi* algorithm averages over multiple gait cycles before detecting TC events to account for the noisy sensor signal. For both approaches, we swapped the definition for the assignment to fit the coordinate system definition (Figure 2a). All algorithms are part of the published Python package EarGait [30].

***Jarchi:*** The algorithm by Jarchi et al. [26] searches for local minima on the dominant oscillation of the AP axis. Subsequently, the minimum of the product of the AP and SI axis in a short interval around each minimum is defined as an IC event. The laterality of an IC event is determined by calculating the mean value of the ML signal for two subsequent IC events. If the mean is greater than the mean of the subsequent IC, it is considered an ipsilateral step. For example, given three subsequent IC s, $IC_{1}$, $IC_{2}$, $IC_{3}$, with $\mu_{1}$ and $\mu_{2}$ being the average of the ML signal from $IC_{1}$ to $IC_{2}$, and $IC_{2}$ to $IC_{3}$, respectively, if $\mu_{1}$ is greater than $\mu_{2}$, $IC_{1}$ is considered as ipsilateral and vice versa. TC events are extracted by finding local minima and maxima on the ML axis. We estimated TCs without averaging over multiple gait cycles, as proposed by the authors, because the detection rate for IC events was significantly worse compared to the other approaches (see Table 2).

***Diao Original:*** Diao et al. [27] first applied a low-pass finite impulse response filter. The dominant oscillation of the SI axis is extracted, and the local minima are considered as ICs. The trend-removed ML axis is used to distinguish between ipsi- and contralateral ICs. If the amplitude of the sample following the IC is greater than the current sample, it is considered IL and vice versa. For example, given an IC at time *t*, the laterality is determined by the following:

(2a) $$\begin{array}{c} acc_{ml\_tr}\left(t+1\right)>acc_{ml\_tr}\left(t\right)\to IC_{IL} \end{array}$$

(2b) $$\begin{array}{c} acc_{ml\_tr}\left(t+1\right)<acc_{ml\_tr}\left(t\right)\to IC_{CL}, \end{array}$$

where $acc_{ml\_tr}$ is the trend-removed ML axis, $IC_{IL}$ refers to an ipsilateral IC, and $IC_{CL}$ to a contralateral IC. The TC depends on the opposite IC and is extracted by finding local minima/maxima on the ML axis with the removed trend.

**Figure 4:**
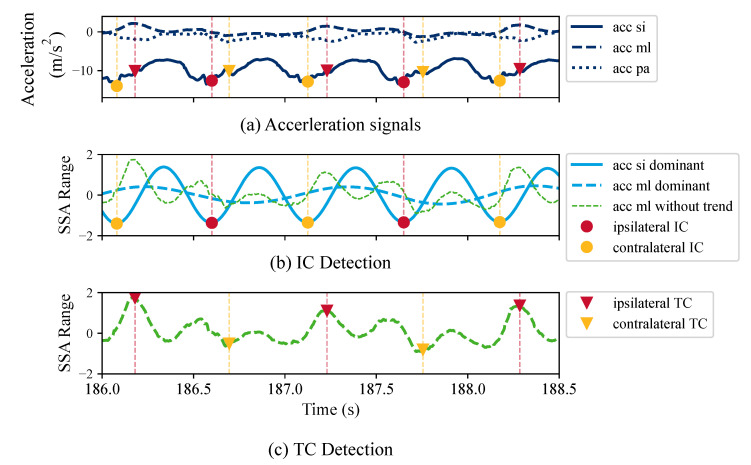
Illustration of the gait event estimation for ear-worn IMUs using the *Diao Improved* algorithm. A Singular Spectrum Analysis (SSA) is used to decompose the acceleration signal. (**a**) Acceleration signals with repetitive gait pattern. (**b**) Dominant oscillation of the superior–inferior (SI) is used to estimate IC events. Laterality of ICs is determined by using the dominant oscillation of the mediolateral (ML) axis. In contrast, the *Diao Original* approach used the trend-removed ML signal (green line) to determine laterality. (**c**) Trend-removed ML signal used to estimate the terminal contacts (TC).

***Diao Improved:*** We propose an improved version of the *Diao Original* algorithm, as we observed that the determination of the laterality of IC events was often incorrect. Instead of deploying the trend-removed ML axis, we used the first dominant oscillation of the ML axis to determine the laterality of each step, ipsilateral or contralateral. Figure 4 depicts the individual steps of the algorithm.

### 2.4. Evaluation

For the SSA, we chose a window length of w=1 s. We observed that some participants walked extremely slow for slow walking compared to others. Outliers, participants with a strong divergent walking speed, were detected for each speed based on a threshold of 2 times the standard deviation (SD). Data of outliers were completely discarded from the evaluation $\left(N_{outliers}=3\right)$ to maintain a balanced data set in terms of the different self-selected walking speeds. Turning steps were discarded using the annotations as described in Section 2.2. For the assessment of different sample frequencies, sensor signals were additionally down-sampled to 50 Hz. To test for significant differences, a one-way ANOVA test with a significance level (*p*-value) of 0.05 was used.

#### 2.4.1. Sensitivity and Laterality Determination

Sensitivity is the ratio of true events and the total number of events that were retrieved by an algorithm. In our case, true events (IC and TC) are determined by the ground truth system, the OMC. To match the detected events to the ground truth events, a tolerance interval of ±0.3 s around each ground truth event was defined. If the detected event is within the interval, it is considered a correctly detected event (true positive).

For all true positives, the correct determination of the laterality, ipsi- or contralateral, was estimated in percent.

#### 2.4.2. Temporal Parameters

Stride, swing, and stance times were calculated based on the definition in [39]. Temporal parameters were defined from two consecutive strides. A stride is defined by the IC of a foot until the next IC of the same foot, referred to as $IC_{start}$ and $IC_{end}$, respectively. Between the two ICs, we expect exactly one TC of the same foot and one IC of the opposite foot ($IC_{contra}$). To obtain the temporal parameters in seconds, each temporal parameter is divided by the sampling rate $f_{s}$.

(3a) $$\begin{array}{cc} StrideTime & =\frac{IC_{end}-IC_{start}}{f_{s}} \end{array}$$

(3b) $$\begin{array}{cc} StepTime & =\frac{IC_{contra}-IC_{start}}{f_{s}} \end{array}$$

(3c) $$\begin{array}{cc} StanceTime & =\frac{TC-IC_{start}}{f_{s}} \end{array}$$

(3d) $$\begin{array}{cc} SwingTime & =\frac{IC_{end}-TC}{f_{s}} \end{array}$$

We calculated the signed error (SE) and the absolute error (AE) for all temporal parameters. Furthermore, we estimated the errors with different granularity: stride-to-stride and straight-walking-bout level. For the latter, parameters were averaged over a straight walking sequence before estimating SE and AE. Each walking bout (Section 2.1) was split into two straight-walking-bout sequences using the turning annotation (Section 2.2). Walking speed often decreases with age, and elderly people may have altered walking patterns. Therefore, we also analyzed the obtained AEs separately for the different self-selected walking speeds and the two study groups, Young and Elderly. Lastly, we evaluated the algorithmic performance for a lower sample rate of 50 Hz because a lower sample rate would be beneficial for embedded devices to reduce battery consumption and computational power.

## 3. Results

### 3.1. Sensitivity and Laterality Determination

To assess the detection rate of the GED algorithms, the sensitivity for IC and TC events was calculated as described in Section 2.4.1. A histogram showing the distances between detected and ground truth events determined the tolerance interval. The results for sensitivity are stated in Table 2. The *Jarchi* algorithm performed considerably worse compared to the other two algorithms, having the lowest sensitivity with about 86%. The sensitivity for *Diao Original* and *Diao Improved* was above 99%. The TC sensitivity for all algorithms was slightly lower than the IC sensitivity.

Table 2 also displays the rate for correct laterality determination for the ICs in percent. The *Jarchi* algorithm had the largest misdetection rate of about 12.6%. Both sensitivity and the laterality determination rate of *Jarchi* were at least 12% lower compared to the other approaches. *Diao Original* had a misdetection rate of about 1.1%, which was further reduced by *Diao Improved* (0.5%). The misdetection rate for TC was not further investigated as the laterality of the TC directly depends on the laterality of the IC.

### 3.2. Temporal Parameters

**Figure 5:**
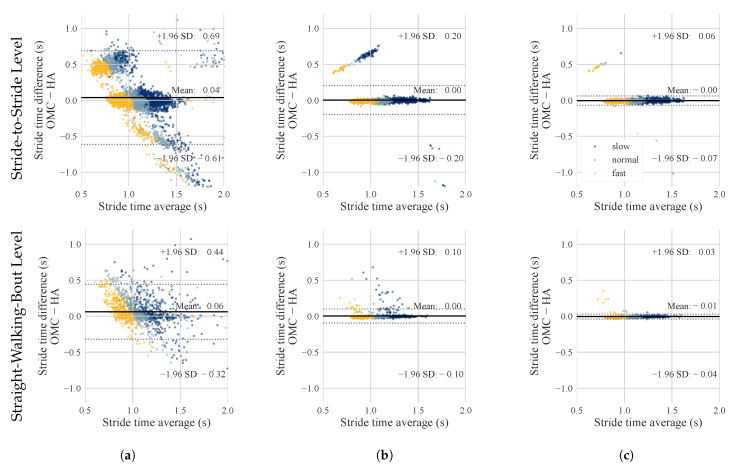
Bland-Altman plots displaying the stride time estimation of ground truth (optical motion capture—OMC) and hearing aid (HA) IMUs on stride-to-stride and straight-walking-bout level for the three implemented algorithms: (**a**) *Jarchi*, (**b**) *Diao Original*, (**c**) *Diao Improved*. On stride-to-stride level, each data point presents a single stride. On straight-walking-bout level, each data point presents the stride time average over the walking sequence. The colors represent the different self-selected walking speeds.

**Figure 6:**
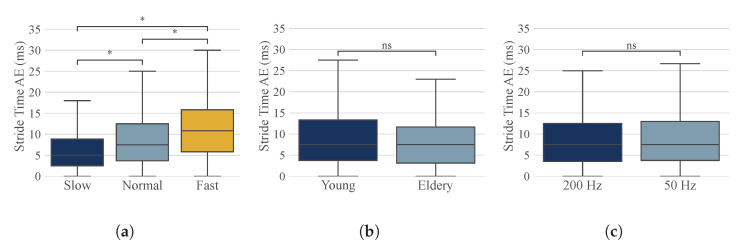
Absolute error (AE) of stride time for the *Diao Improved* algorithm for the different (**a**) walking speeds, (**b**) study groups, and (**c**) sample rates. The AE for walking speeds differs significantly (*; one-way ANOVA, p≤0.05), but study groups and sample rates do not differ significantly (ns—not significant; p>0.05).

The estimated temporal gait parameters for the three different algorithms were compared to the ground truth (OMC) on stride-to-stride and straight-walking-bout level. The *Jarchi* algorithm had the highest errors and also showed the largest spread for the stride time error (Table 3, Figure 5). The absolute stride time error for *Jarchi* (170 ± 288 ms, stride-to-stride level) was about 7 times higher compared to the other algorithms (Table 3). *Diao Original* achieved the lowest stride time error of 12 ± 32 ms (stride-to-stride level). The stride time error of *Diao Original* was approximately twice as high (24 ± 100 ms), with a considerably higher standard deviation. The same applied to the straight-walking-bout level. In terms of the stride-level, *Diao Original* showed an accumulation of data points at around 0.5 s error (Figure 5b), which was also visible to a lesser degree for *Diao Improved*. For step time and stride time, the error decreased for the straight-walking-bout level, while the error remained comparable for stance and swing time (Table 3). For the *Diao Improved* algorithm, absolute stride time error was further analyzed with respect to walking speed, study population, and sampling frequency (Figure 6, Table A1 in Appendix A). We observed a significant difference for the different walking speeds, with slow walking having the smallest and fast walking the largest AE. We observed no significant difference for the different study groups and sample rates.

## 4. Discussion

This work contributes a validation for gait event detection and temporal gait parameter estimation using ear-worn IMUs integrated into a conventional HA. We compared two existing algorithms, Jarchi et al. [26] and Diao et al. [27], and proposed an improved version of the latter to enhance laterality determination and, therefore, stride time estimation. The algorithms were validated against an OMC-based gold standard. We assessed the sensitivity, laterality determination, and temporal gait parameters for different walking speeds, study populations, and sampling frequencies. The implemented algorithms and data processing functionalities are published as an open-source Python package called EarGait [30].

### 4.1. Comparison of Algorithms

The *Jarchi* algorithm performed considerably worse in all categories compared to the other two algorithms. We observed that two or multiple consecutive IC events were often assigned to the same side (IL or CL). Furthermore, the low laterality rate and the large scatter in the Bland–Altman plot (Figure 5a) prove that the *Jarchi* algorithms struggled to differentiate between IL and CL events, which directly leads to a large error for the stride time. The diagonal bias in the Bland–Altman plot results from the large deviations of the calculated stride time from the actual stride time by the OMC system (over- and underestimations). In their work, Jarchi et al. [26] reported a considerably lower error of 18 ± 22 ms. It seems as if their approach performed well for their ear-worn sensor position but did not generalize well for the HA’s sensor position. A reason for that might be that the orientation of the ear-mounted sensor might slightly differ, influencing the axis alignment.

We also added gravity alignment to compensate for slightly varying sensor positions. All in all, when using the hearing aid’s integrated IMUs, the *Jarchi* algorithm is not able to correctly detect gait events and estimate temporal parameters with high accuracy.

Opposed to *Jarchi*, the overall performance of *Diao Original* and *Diao Improved* were in a similar range, with *Diao Improved* having the best performance in all categories. The IC sensitivity for both algorithms was above 99%. Overall, TC sensitivity was slightly lower compared to the IC. We expected that, as the TC detection depends on the ICs. The TC sensitivity of *Diao Original* was 4% less, and the laterality determination rate was also slightly lower compared to *Diao Improved*. With a stride time error of 24 ± 100 ms, the error for the *Diao Original* algorithm was in the same range although slightly less, as reported by the authors (28 ms) [27]. The sensitivity and laterality assignment rate can not be discussed in relation to the results of the original publications, as those papers did not address these metrics.

### 4.2. Laterality Enhancement

Laterality is important for asymmetry estimation, which can be a meaningful parameter for fall prediction [40] or detecting mild cognitive impairments [41]. Furthermore, the correct laterality determination is important for stride time estimations. The difference in the laterality determination rate of 0.6% between *Diao Original* and *Diao Improved* seems small; however, it had a major effect on the stride time estimation, as the AE of *Diao Original* was about twice the error of *Diao Improved*. The Bland–Altman plot also shows a larger scatter for *Diao Original* (Figure 5b). The outliers are located around 0.5 s, which corresponds to about half a stride; hence, one step or 50%. These outliers are due to an incorrect laterality determination, and the 50% error results in a linear dependency for the absolute error of stride time. Note that the step error for *Diao Original* and *Diao Improved* was equal. Step time estimation depends on the detection of ICs but not on the corresponding side. The detection of ICs is equal for the *Diao Original* and *Diao Improved* algorithms. The difference is the underlying method for laterality assignment.

One might argue that an alternating step sequence is obvious and could be enforced within an algorithm. However, the TC detection directly depends on the laterality of the IC, and hence a correct distinction for most steps is essential. Furthermore, enforcing alternating step sequences would only lead to improvement, with the strict assumption that all ICs had been detected. However, in reality, some ICs will be missed, making enforced alternating step sequences a large error source. Alternatively, for practical real-world applications in which only the mean stride time over a gait sequence is of interest, the SD could be reduced by adding a postprocessing step that neglects sequences with non-alternating IL and CL steps for stride time estimation. In an additional experiment applying this postprocessing step, the SD was halved; however, the mean error remained the same.

### 4.3. Temporal Parameters

For all evaluation metrics, the *Diao Improved* performed best with an absolute stride time error of 12 ± 32 ms. The improved version of the *Diao Original* algorithm was developed to reduce the number of incorrect IL and CL assignments. The stride time error was halved compared to the original version, and the SD was reduced to one-third. As well as stride time, we also assessed stance and swing time, which showed a similar error in terms of magnitude but opposite in direction (Table 3). In contrast to step and stride time, the stance and swing phases depend on both gait events, IC and TC. It might be possible that the OMC and the IMU-based system detect slightly different time points for TC events leading to a systematic offset. For applications in which stance and swing are of importance, further investigations regarding TC detection should be considered. The error of about 77 ms corresponds to about 8% of a gait cycle, which is higher than the natural variations for healthy people [42].

In comparison to the lower-trunk approach introduced by Zijlstra et al. [43], this study demonstrated a similar range for the stride time error (2–15 ms dependent on the walking speed). Trojaniello et al. [44], who also used lower-trunk-worn IMUs, reported a considerably lower stride time error of 1 ± 11 ms. It should be noted that the walking speed in [44] was in between the slow and normal walking speed of our work. For slower walking speeds, the error of this work was also significantly lower, and hence, within a similar range as that reported by Trojaniello et al. (Table A1). Compared to foot-worn IMUs as proposed by Rampp et al. [39], the stride time error of our method is smaller; however, stance and swing time errors are twice as high, although Rampp et al. validated their approach for Parkinson’s patients, which might have contributed to the higher error reported there. In [45], shank-worn sensors were used achieving a stride time AE of 6–9 ms, which is lower compared to our approach (12 ms). In general, we expected leg-worn IMUs to be more accurate because they are much closer to the origin of movement, and the head has more additional movement artifacts. However, the head position is more convenient for home monitoring than other single or leg-worn sensor approaches, as the sensors can easily be integrated into devices used on a daily basis, such as glasses, earbuds, or HAs.

In practice, users or clinical experts may not be interested in each individual stride-wise parameter but rather in the average stride parameters over a certain period of time, for instance, a straight walking bout. Therefore, the error for temporal parameters was also calculated on a straight-walking-bout level. The overall error decreases for stride and step time, although not significantly.

Due to different walking patterns for the elderly, it is important to validate our algorithms for elderly people as well. The proposed improved algorithm is reliable for different study populations; however, it is sensitive to walking speed (Figure 6), having the smallest error for slow walking. Studies have shown that gait speed often decreases with age. As elderly people are the leading target group for HA needs, slow walking was of particular interest. Furthermore, to be able to run on a small embedded device such as a hearing aid, computational complexity needs to be considered. Reducing the sampling frequency to 50 Hz did not change the results. With respect to small embedded devices, a lower sample rate would naturally reduce the overall computational complexity and power consumption of the sensors. Even with a reduced sample rate, the proposed algorithm *Diao Improved* is not yet applicable in real time. However, it may serve as inspiration for embedding in a small processing unit such as an HA enabling continuous and long-term analysis.

### 4.4. Usability

A hearing aid would be ideal for home monitoring, as it is already part of the user’s everyday life, in contrast to most other approaches, such as, for instance, Jarchi et al. [26] and Diao et al. [27], who built a system in which the user would need to wear an extra sensor. Furthermore, no specialist for sensor positioning is needed, as hearing aid users are familiar with inserting the hearing aid by themselves. Smartwatches, which are capable of performing similar gait analysis, are nowadays also part of many people’s life, and about 17% of elderly people own a smartwatch [46]. Compared to that, hearing aids have a greater potential as about every second elderly person needs a hearing aid. However, in reality, many hearing losses go untreated, and only about 15–20% of people with hearing loss are provided with hearing aids [47]. The higher number of elderly wearing a smartwatch proves that the elderly, in general, are interested in using health trackers. Hearing aids with integrated health trackers might increase the attractiveness of hearing aids and provide additional motivation for using them.

### 4.5. Limitations and Future Work

One limitation of the proposed algorithm is that it is only applicable to gait signals. A low-cost preprocessing algorithm that detects gait sequences within a continuous signal is needed. Additionally, data were recorded in a controlled laboratory environment with a limited walking path length. This controlled setting may have influenced participants’ behavior and led to many acceleration, deceleration, and turning steps. Therefore, it is crucial to investigate the reproducibility of the algorithm in real-world scenarios. Moreover, it should be acknowledged that the participants were all healthy individuals without mobility restrictions. Although the SSA-based algorithm performed very well for steady walking, its performance for unsteady gait remains to be explored and evaluated. Furthermore, audiological aspects influencing gait patterns could be analyzed with the help of this method. Future research should also address other gait parameters or digital mobility outcomes, such as step length, cadence, gait asymmetry, or turning performance. As well as gait analysis, hearing aid integrated IMUs could be used for other health monitoring approaches, such as human activity recognition or sedentary movement analysis, transforming a conventional hearing aid into an extensive physical health tracking system [7].

## 5. Conclusions

This work contributes a validation of gait event algorithms and temporal parameter estimation for ear-worn IMUs integrated into HAs. We published an open-source Python package called *EarGait* [30] that includes data loading functionalities, event detection algorithms, and temporal parameter estimation. Our analysis showed that hearing aid integrated sensors are suitable for estimating gait events and temporal parameters with high precision. The improved algorithm proposed in this work enhanced the laterality determination of gait events and, therefore, stride time estimation. Our work covers the first but crucial step in a comprehensive gait analysis pipeline since most subsequent algorithms, such as step length estimation, rely on accurately detected events. Considering the increasing prevalence of earables such as hearing aids and earbuds in people’s everyday life, our system holds the potential for seamless integration into users’ daily routines. As gait impairments and hearing loss increase with age, a mobility assessment system embedded into an HA would be particularly beneficial. With advances in hardware power consumption, hearing aids may facilitate unobtrusive and convenient gait monitoring in a person’s natural environment without an additional device.

## Figures and Tables

**Table 1:** Participants’ characteristics and self-selected walking speeds for the recorded data set containing two groups: Young (N = 27, 18–59 years) and Elderly (N = 21, 60–83 years). Walking speed is based on the reference system (optical motion capture system).

Characteristic	Young	Elderly
Gender (m/f)	11/16	10/11
Age (years)	31 ± 11	71 ± 6
Height (cm)	172 ± 8	167 ± 8
Weight (kg)	64 ± 9	72 ± 10
Hearing loss * (yes/no)	0/27	9/12
Self-selected walking speed (m s^−1^)		
slow **	1.06 ± 0.15	1.09 ± 0.14
normal **	1.38 ± 0.07	1.36 ± 0.12
fast **	1.85 ± 0.16	1.72 ± 0.14

* Degree of hearing loss: slight–moderate (World Health Organization). ** Significant difference in self-selected walking speed between Young and Elderly. One-way ANOVA: p≤0.05.

**Table 2:** Sensitivity for initial (IC) and terminal contacts (TC) and laterality determination rate (in %) for IC events.

	*Jarchi*	*Diao Original*	*Diao Improved*	
	200 Hz	200 Hz	200 Hz	50 Hz
IC (%)	85.8	99.1	99.8	99.8
TC (%)	71.2	93.5	97.5	97.4
Laterality Determination (%)	87.4	98.9	99.5	99.4

**Table 3:** Absolute (AE) and signed error (SE) of temporal parameters for the three algorithms aggregated on stride-to-stride and straight-walking-bout level. Error metrics are provided as mean ± standard deviation. The best results for stride and step time are highlighted in **bold**.

		Stride-to-Stride Level				Straight-Walk Level			
		*Diao Improved*		*Diao Original*	*Jarchi*	*Diao Improved*		*Diao Original*	*Jarchi*
		200 Hz	50 Hz	200 Hz	200 Hz	200 Hz	50 Hz	200 Hz	200 Hz
AE	Stride time (ms)	**12 ± 32**	14 ± 36	24 ± 100	170 ± 288	**10 ± 16**	11 ± 21	17 ± 47	129 ± 158
	Step time (ms)	**9 ± 12**	11 ± 13	**9 ± 12**	64 ± 94	**5 ± 8**	5 ± 7	**5 ± 8**	34 ± 55
	Stance time (ms)	76 ± 34	76 ± 36	76 ± 33	201 ± 247	71 ± 29	71 ± 29	72 ± 29	160 ± 161
	Swing time (ms)	78 ± 33	78 ± 34	78 ± 32	154 ± 144	74 ± 27	74 ± 27	75 ± 27	109 ± 72
SE	Stride time (ms)	−4 ± 33	−4 ± 39	3 ± 103	37 ± 333	−6 ± 18	−5 ± 23	2 ± 50	61 ± 195
	Step time (ms)	−3 ± 15	−3 ± 17	−3 ± 15	−7 ± 113	−3 ± 8	−3 ± 8	−3 ± 8	−10 ± 64
	Stance time (ms)	74 ± 38	74 ± 41	75 ± 36	−50 ± 314	70 ± 31	70 ± 31	71 ± 31	−46 ± 223
	Swing time (ms)	−76 ± 36	−76 ± 38	−77 ± 35	72 ± 198	−74 ± 28	−74 ± 28	−74 ± 29	66 ± 113

## Data Availability

The data presented in this study are available upon reasonable request from A.-K.S. The participants of the presented study did not consent to the publication of their sensor data in open repositories, in accordance with European data protection laws.

## Abbreviations

The following abbreviations are used in this manuscript:

CL	Contralateral
HA	Hearing aid
IC	Initial contact
IL	Ipsilateral
IMU	Inertial measurement unit
AE	Absolute error
ML	Medial–lateral
OMC	Optical motion capture
PA	Posterior–anterior
SD	Standard deviation
SE	Signed error
SI	Superior–inferior
SSA	Singular Spectrum Analysis
TC	Terminal Contact

## Appendix A

**Table A1:** Absolute (AE) and signed error (SE) of temporal parameters for the different self-selected walking speeds and the different study groups. Temporal parameters were aggregated on stride-to-stride and straight-walking-bout level. Error metrics are provided as mean ± standard deviation.

		Stride-to-Stride Level						Straight-Walk Level				
		Slow	Normal	Fast	Young	Elderly		Slow	Normal	Fast	Young	Elderly
AE	Stride time (ms)	9 ± 13	11 ± 39	16 ± 43	11 ± 26	12 ± 36		7 ± 6	9 ± 8	14 ± 27	10 ± 13	9 ± 19
	Step time (ms)	10 ± 8	8 ± 18	8 ± 7	9 ± 12	9 ± 12		3 ± 3	5 ± 12	6 ± 4	5 ± 8	5 ± 8
	Stance time (ms)	88 ± 38	73 ± 26	58 ± 26	77 ± 34	75 ± 33		87 ± 32	70 ± 22	54 ± 20	71 ± 27	71 ± 30
	Swing time (ms)	90 ± 38	77 ± 26	62 ± 25	79 ± 33	77 ± 33		89 ± 30	75 ± 21	59 ± 18	75 ± 25	74 ± 28
SE	Stride time (ms)	−2 ± 16	−6 ± 40	−6 ± 45	−6 ± 28	−3 ± 37		−3 ± 8	−7 ± 9	−7 ± 29	−7 ± 15	−4 ± 20
	Step time (ms)	−1 ± 12	−3 ± 19	−5 ± 9	−4 ± 14	−2 ± 15		−1 ± 4	−3 ± 13	−6 ± 5	−4 ± 8	−3 ± 8
	Stance time (ms)	86 ± 43	72 ± 28	55 ± 31	76 ± 38	73 ± 38		85 ± 36	70 ± 23	53 ± 22	71 ± 28	69 ± 34
	Swing time (ms)	−87 ± 43	−76 ± 28	−61 ± 28	−78 ± 35	−74 ± 37		−87 ± 35	−75 ± 21	−59 ± 18	−75 ± 25	−72 ± 31
